# Creatine-Kinase- and Exercise-Related Muscle Damage Implications for Muscle Performance and Recovery

**DOI:** 10.1155/2012/960363

**Published:** 2012-01-11

**Authors:** Marianne F. Baird, Scott M. Graham, Julien S. Baker, Gordon F. Bickerstaff

**Affiliations:** School of Science, University of the West of Scotland, Paisley PA1 2BE, UK

## Abstract

The appearance of creatine kinase (CK) in blood has been generally considered to be an indirect marker of muscle damage, particularly for diagnosis of medical conditions such as myocardial infarction, muscular dystrophy, and cerebral diseases. However, there is controversy in the literature concerning its validity in reflecting muscle damage as a consequence of level and intensity of physical exercise. Nonmodifiable factors, for example, ethnicity, age, and gender, can also affect enzyme tissue activity and subsequent CK serum levels. The extent of effect suggests that acceptable upper limits of normal CK levels may need to be reset to recognise the impact of these factors. There is a need for standardisation of protocols and stronger guidelines which would facilitate greater scientific integrity. The purpose of this paper is to examine current evidence and opinion relating to the release of CK from skeletal muscle in response to physical activity and examine if elevated concentrations are a health concern.

## 1. Introduction

CK is a compact enzyme of around 82 kDa that is found in both the cytosol and mitochondria of tissues where energy demands are high. In the cytosol, CK is composed of two polypeptide subunits of around 42 kDa, and two types of subunit are found: M (muscle type) and B (brain type). These subunits allow the formation of three tissue-specific isoenzymes: CK-MB (cardiac muscle), CK-MM (skeletal muscle), and CK-BB (brain). Typically, the ratio of subunits varies with muscle type: skeletal muscle: 98% MM and 2% MB and cardiac muscle: 70–80% MM and 20–30% MB, while brain has predominantly BB. In mitochondria there are two specific forms of mitochondrial CK (Mt-CK): a nonsarcomeric type called ubiquitous Mt-CK expressed in various tissues such as brain, smooth muscle, and sperm, and a sarcomeric Mt-CK expressed in cardiac and skeletal muscle [1].

CK also occurs as macroenzymes. Macro-CK type 1 is a complex of CK (most often CK-BB) and immunoglobulin (most often IgG) and is typically greater than 200 kDa in size. Macro-CK type 2 is a polymer of Mt-CK with a molecular mass of greater than 300 kDa [2]. These forms of CK are expressed during disease and/or dysfunction, for example, macro-CK 1 is associated with cardiovascular and autoimmune disease and macro-CK 2 with cancer. CK catalyzes the reversible phosphorylation of creatine to phosphocreatine and of ADP to ATP [3, 4], and as such it is important in regeneration of cellular ATP:

(1) $$\text{Phosphocreatine}+{\text{MgADP}}^{-}+{\text{H}}^{+}\Leftrightarrow {\text{MgATP}}^{2-}+\text{creatine}$$

**Figure 1:**
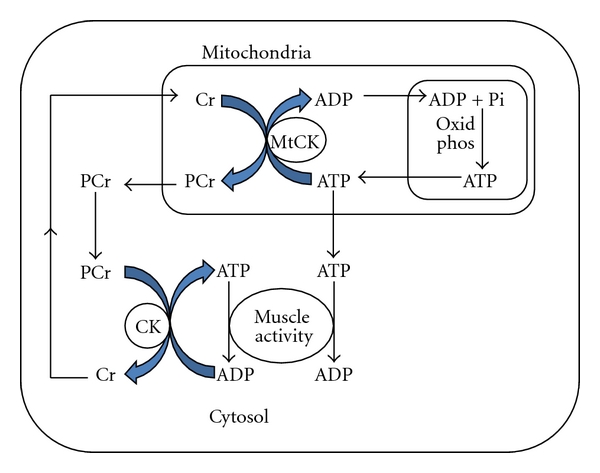
Phosphocreatine (PCr) circuit showing the rephosphorylation of creatine (Cr) in mitochondria using ATP derived from oxidative phosphorylation (oxid phos) and subsequent use of mitochondrial PCr by cytosolic creatine kinase (CK) to resupply ATP for muscle activity, adapted from Saks [5].

CK forms the core of an energy network known as the phosphocreatine (PCr) circuit (see Figure 1). In this circuit, the cytosol isoenzymes are closely coupled to glycolysis and produce ATP for muscle activity. The MtCK version is closely coupled to the electron transport chain and can use mitochondrial ATP to regenerate PCr, which readily returns to the cytosol to resupply cytosolic PCr. This shuttle system is critical for the production and maintenance of energy supply and is involved in the metabolic feedback regulation of respiration [5]. It is unsurprising, therefore, that skeletal muscle has high levels of CK that can account for as much as 20% of the soluble sarcoplasmic protein in specific muscles.

Until the mid-1990s, determination of serum CK levels was a key tool in the diagnosis of myocardial infarction (MI) in patients presenting with chest pain in emergency departments. Subsequently, the diagnostic role has been replaced, to a certain extent, by the muscle protein troponin. However, raised levels of serum CK are still closely associated with cell damage, muscle cell disruption, or disease. These cellular disturbances can cause CK to leak from cells into blood serum [6]. Measurement of serum CK activity and determination of isoenzyme profiles are still an important indicator of the occurrence of muscle cell necrosis and tissue damage due to disease or trauma [3]. 

There has been extensive discussion in the literature regarding the significance of raised levels of serum CK following physical exercise in relation to degrees of muscle cell damage or disturbance. While the reason for release of CK into the circulation is clear in cases such as MI, it is less clear why low- to moderate-intensity physical exercise should also result in release of CK into blood serum. It is certainly confusing that resistance training elicits the greatest release of CK and at the same time provides the best route for muscle hypertrophy. Myofibrillar CK-MM is bound to the M-line of the sarcoplasmic reticulum of myofibrils and is also found in the space of the I-band sarcomeres providing support for muscle energy requirements [7]. Thus, the enzyme is normally confined to the muscle cell so the question arises: do raised levels of CK following a period of exercise represent a degree of actual muscle damage and loss of muscle cell integrity, or is there some other molecular explanation that is not permanent cell damage, but a temporal disturbance or disruption to muscle processes? A greater understanding of this issue could have significant implications for exercise strategy and training programme design (performance and recovery). This is true, not only for athletic populations but also for individuals who participate in strenuous exercise as part of their lifestyle. In this paper, we examine current evidence and opinion relating to the release of CK from skeletal muscle tissue into blood serum in response to muscle exercise.

## 2. Muscle Response to Exercise

Each mature skeletal muscle fibre is a single cell fused from around 100 myoblasts; following fusion, myoblasts lose cell division capacity. Skeletal cell numbers are established before birth. These cells are designed to last a lifetime and are not subject to turnover and recycling processes that occur in many other cell types. Growth in muscle mass happens in magnitude only (hypertrophy via growth hormone and testosterone). While hypertrophy is readily reversible (atrophy), loss of muscle cell numbers as a result of damage would be progressively more serious. Muscles are arranged in bundles of various grade and strength that allow for variable muscle force to suit each need relating to maximal or minimum contractions. Motor units consisting of nerve fibre and associated groups of muscle fibres are recruited as required by nerve stimulation. For stronger contractions more motor units are recruited.

Peripheral muscle fatigue is generally viewed, as a result of insufficient energy and availability of key metabolites that enable contracting muscles to meet increased energy demand. Lack of energy and metabolites will result in motor groups that are unable to fulfill the required workload. Thus, the control of peripheral systems is dependent on the prevailing local metabolism in a motor unit, whereas, in the central model of muscle fatigue, neuromuscular mechanisms aim to preserve overall integrity of the system by mechanisms such as motor unit derecruitment. Golgi tendon organs (GTOs) monitor the tension produced by contraction to prevent excess forces by continuous feedback to the central nervous system (CNS). Thus, the CNS is informed by collective feedback mechanisms that include chemical, mechanical, and cognitive cues. The significance of each of these cues will depend on duration and power requirements of muscular activity. While GTO feedback can be overridden by cognitive processes in the CNS, to allow an athlete to increase performance, it is likely that local peripheral systems can prevent the level of excess muscle contraction that could result in failure or damage.

**Figure 2:**
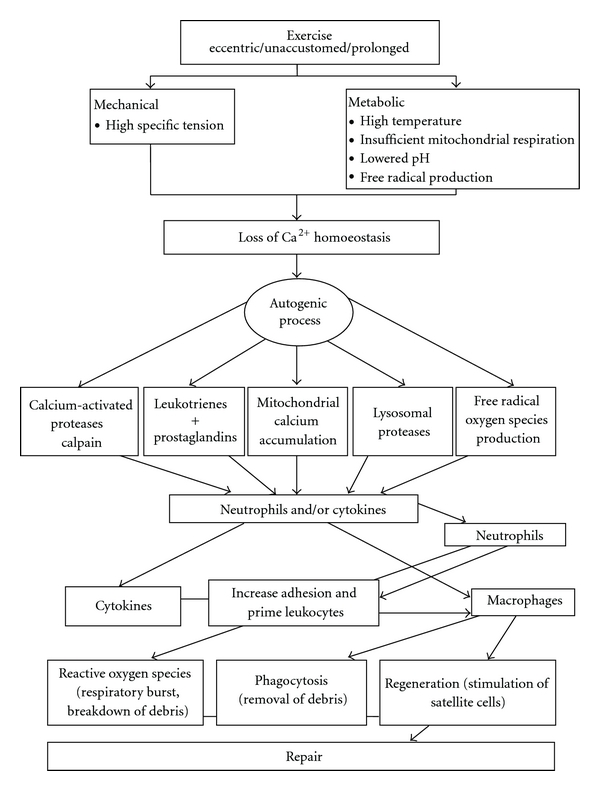
Theoretical model of muscle damage and repair cycle reproduced from Kendall and Eston [11].

**Figure 3:**
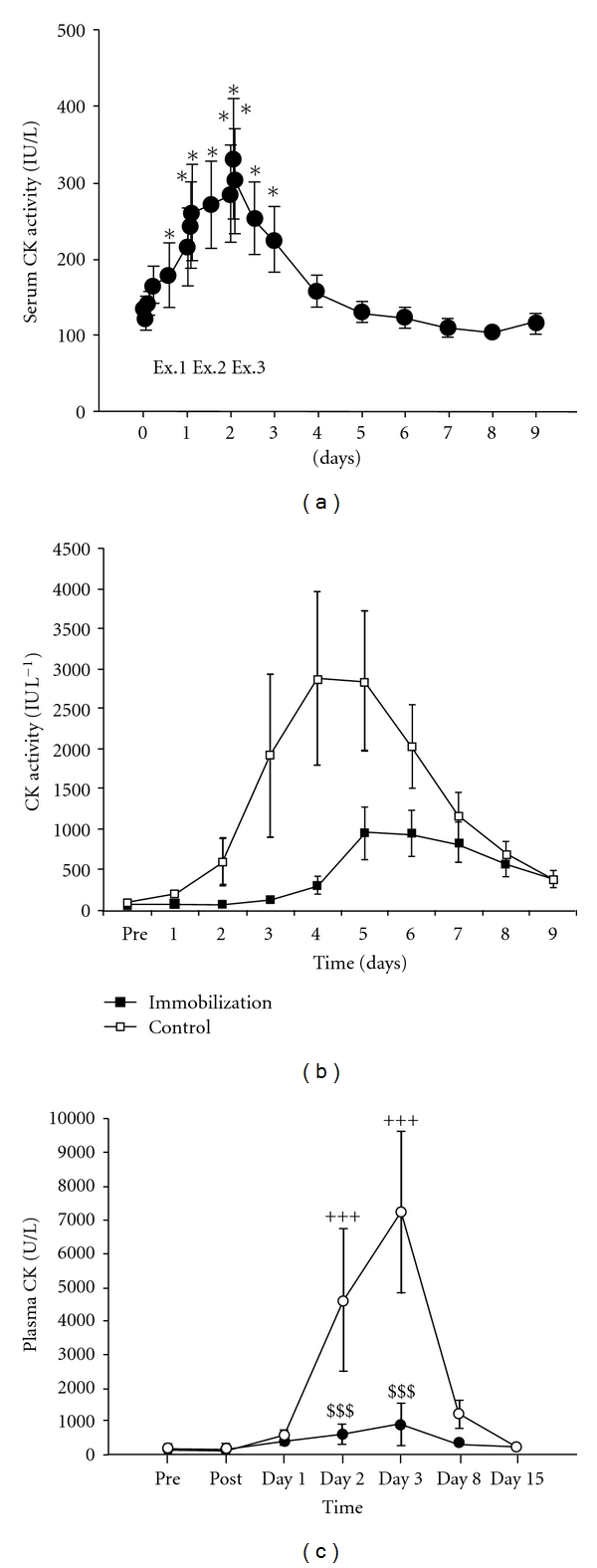
(a) Changes in serum creatine kinase (CK) activity during 90-minute cycling exercise on three consecutive days (Ex1, Ex2, and Ex3), reprinted from Totsuka et al. with permission from American Physiological Society [6]. (b) Creatine kinase (CK) response to eccentric exercise between immobilisation and control group. PRE refers to the baseline period before exercise. Days 1–4 represent the 4-day immobilization and days 5–9 are the recovery period. Reprinted from Sayers and Clarkson [4]. (c) Creatine kinase (CK) activity in women and in men before (pre), immediately after (post), and 15 days after step exercise. +++ Significant difference from preexercise level (*P* < 0.001). $$$ Significant difference between men and women (*P* < 0.001), reprinted from Fredsted et al. [12].

Unaccustomed exercise, particularly eccentric muscle contractions, initiates mechanical muscle damage of varying degrees [8]. Metabolic muscle disturbance is thought to result in release of cellular components through a cascade of events, which begin with depletion of ATP and result in the leakage of extracellular calcium ions into intracellular space, due to both Na-K-ATPase and Ca^2+^-ATPase pump dysfunction. Intracellular proteolytic enzyme activity can increase and promote muscle protein degradation and augmented cell permeability, which allows some cell contents to leak into the circulation [9, 10]. The process of mechanical and metabolic initiated muscle disruption is not entirely understood; it is thought to consist of a complex range of events involving increased oxidative stress, inflammatory and immune responses. Loss of cell myofibre proteins into the blood may occurs at several stages along this continuum (see Figure 2). In most cases, isolated mild to moderate damage in otherwise healthy individuals does not appear to cause further problems, and many studies have demonstrated that the body is capable of clearing released muscle components back to baseline levels within 7–9 days [4, 6] (see Figures 3(a)–3(c)).

Factors such as temperature extreme, alcohol abuse, or sporadic strenuous exercise, for example, ultra marathons, can result in more severe disturbance and may require medical intervention to prevent permanent renal damage, primarily due the nephrotoxic effects of myoglobin [9]. Some individuals are found to have high levels of serum CK compared to other similar individuals when exposed to the same exercise protocol (including moderate exercise) even when main comparability factors such as gender, age, and training status are accounted for in data analysis. In some cases, this variability may indicate an underlying myosis, but in many other cases the cause is unknown [7]. There appears to be no established link between habitual exercise or acute high-intensity eccentric exercise and raised incidence of kidney dysfunction or muscle disorder in normal healthy individuals, even in the presence of CK levels >20.000 U/L^−1^. The contribution of additional factors such as genetic disposition, environmental conditions, or disease may increase the risk of exertional rhabdomyolysis resulting in acute renal failure [13] (see Table 1).

Individuals who regularly participate in high-volume, intense exercise, tend to have significantly raised base levels of CK compared to sedentary and moderately exercising individuals [14]. Raised levels of serum CK were also found in regularly exercising pre-menopausal women compared to similar sedentary individuals [15]; this suggests that CK flux into the serum is a natural and normal reaction to regular exercise.

## 3. Clinical Significance of Raised Serum CK

Base levels of serum CK in general populations are variable 35–175 U/L [16] with ranges from 20 to 16,000 U/L, and this wide range reflects the inconsistent occurrence of subclinical disorders and minor injury, genetic factors, physical activity status, and medication [17].

In examples of rhabdomyolysis (clinically diagnosed muscle damage) CK levels have been found at 10,000–200,000 U/L and as high as 3 × 10^6^ U/L [18]. Such levels clearly signal strong disturbance or disintegration of striated muscle tissue with concomitant leakage of intracellular muscle constituents into the circulation. In the absence of specific myocardial or brain infarction, physical trauma, or disease, serum CK levels greater than 5,000 U/L are generally considered to indicate serious disturbance to muscle [10]. It has been recommended acceptable upper limits of normal CK levels be increased by 1.5 times the present limits and that muscle biopsy investigations are only necessary when levels are ≥3 times greater than upper limits and in the absence of exercise induced explanations [19]. However, there is no universally agreed or accepted standard. There are many possible reasons for a diagnosis of rhabdomyolysis and accompanying raised CK levels (see Table 1). Most of the conditions in Table 1 can be attributed to disruption of cells/cell membranes, localised hypoxia, and depletion of energy and disruption of electrolyte balance. Raised levels of macro-CK tend to be associated with disease, though they can also be present in apparently healthy individuals [20].

## 4. CK Marker for Muscle Damage or Performance Capacity

There is extensive debate in the literature concerning the reliability of serum CK level as a marker of muscle damage. Serum CK determinations are normally initial measures of enzyme activity in blood at the time of sampling, and timeline profiles are mostly set and influenced by the requirements of diagnosis of MI and stroke rather than any exercise influence. The mechanism(s) by which CK is cleared from the blood has not been fully elucidated, and it is likely that observed serum CK levels reflect complex interactions associated with energy status and scale of muscle disturbance. Thus, measured serum CK will reflect relative amounts of CK released, degree of enzyme activity of released CK, and the rate of clearance of CK from the serum [15].

 In general terms, high serum CK in some ethnic groups may reflect a genetic condition of naturally increased levels of CK muscle tissue activity, which is not related to exercise frequency or muscle disturbance [21]. It has been proposed that higher than normal levels of tissue CK activity may augment the availability of cellular energy and improve myofibril contraction responses [21]. Therefore, high levels of serum CK, in the absence of muscle damage or other pathological conditions, may reflect the level of enzyme tissue activity of the individual.

Serum CK levels alone may not provide a fully accurate reflection of structural damage to muscle cells [22, 23]. Some studies have reported that serum CK levels were affected by hydration status prior to eccentric exercise and varied within subject groups of comparable male volunteers, whilst muscle biopsies revealed similar ultrastructure damage to Z-band muscle fibres. Muscle soreness did not differ between groups [24]. Biopsies are specific only to a small area of investigation and therefore may not represent the universal extent of damage to the muscle groups exercised. Indeed, the biopsy procedure may itself cause damage to muscle fibres. Other additional indirect indices of muscle damage such as magnetic resonance studies and assessment of delayed onset muscle soreness (DOMS) (which include reduced muscle force post exercise, swelling, perception of pain, and reduced range of movement (ROM)) have been utilized in many studies [25, 26] as have other blood chemical markers of inflammation and stress [27, 28]. These additional measures can assist in quantifying and substantiating muscle disturbance parameters.

## 5. Exercise Type and Muscle Disruption

Low-intensity (LI) exercise (50% of maximal isometric strength) induced less magnitude of muscle damage and decline in muscle performance than maximal eccentric exercise when the same amount of sets and reps were performed (3 × 10 reps) [29]. Although sets and reps were matched in this study, work volume was not standardised.

Dynamic concentric and eccentric leg extensions were performed by 21 untrained men and women [30]. Higher-intensity (70% 10 RM and 90% 10 RM) exercise, whilst maintaining a constant 150 reps, elicited greater serum CK, glutamic oxaloacetic transaminase, and serum lactate dehydrogenase levels than lower-intensity (35% 10 RM) exercise. Similarly, when duration of work was increased by performing a greater number of reps, whilst maintaining intensity at 70% 10 RM, serum indices of muscle damage were higher. Therefore, as the volume of exercise performed increased metabolic demands, as might be anticipated, indices of muscle damage were augmented. Interestingly, however, when total work performed was equalized by inversely varying intensity and duration, the greatest rise in serum enzyme levels occurred in the highest-intensity exercise with the shortest duration (80% 10 RM, 170 reps) than following the session with longer duration and lower intensity exercise (30% 10 RM, 545 reps). These results suggest that the magnitude of exercise intensity has greater influence on cellular response to exercise-induced muscle damage than the duration. Another research [31] compared equal volumes of high- and low-intensity eccentric leg extensions on untrained subjects. In this study, work volume was equalised using an isokinetic dynamometer. The authors concluded that there was no significant difference in muscle disturbance indicators (except at 24 hrs). However, high-intensity (HI) exercise did elicit larger declines in muscle performance and a slower recovery. This may be due to a greater recruitment of type II muscle fibres in high-intensity eccentric exercise, which have been found to be more susceptible to disruption compared to type I [23, 32]. Serum CK levels were higher with high intensity, but not significantly. Subjective measurement of pain and ROM measurements showed no significant difference between groups. In this study, equal volumes of work result in similar indices of muscle disruption, but with less decrement in muscle performance, and greater recovery with low intensity compared to high intensity. The use of leg extensions in the latter study compared to elbow flexion in Nosaka and Newton's study [29] may have contributed to the variations in muscle disturbance indices between the two studies. There is evidence to suggest that the degree of muscle damage is greater in elbow flexion compared to knee extension [26]. However, both studies did agree in their findings concerning greater declines in muscle performance after HI compared to LI.

Trained soldiers (age 19.1 ± 1.8 yrs) were randomly assigned to one of four experimental groups using a bench press protocol (*n* = 8, 50% 1 RM, *n* = 7, 75% 1 RM, *n* = 7, 90% 1 RM, *n* = 7, 110% 1 RM, and a control group *n* = 6). There was no significant difference in the total volume of exercise among the groups. All subjects showed a significant (*F* = 2.40, *P* < 0.03) increase in postexercise CK activity. The highest values occurred at 24, 48, or 72 h; however, no significant (*P* = 0.2) difference was found between groups, although there was a large variability between subjects. There was no significant difference in muscle soreness between groups (*P* = 0.39). The 110% 1 RM had a significantly higher (*P* < 0.05) prostaglandin E_2_ (PGE_2_) than the other groups at 24 and 48 h after exercise [33].

This study also concludes that volume of exercise rather than intensity determines the level of muscle damage; however, the subjects in the 50, 75, and 90% groups executed eccentric and concentric actions, whereas the 110% group only performed eccentric contractions. This may have influenced the magnitude of muscle damage in the 110% group. Total volume of exercise was determined from a calculation (total volume = number  of  sets × number  of  repetitions × load (kg)); therefore the calculated volume may not have been determined as accurately as the isokinetic dynamometer protocol.

 The higher levels of PGE_2_ in the 110% 1 RM group suggest a greater magnitude of inflammation at 24 and 48 h compared to the other groups. Muscle force measurement may have further evidenced any variations in strength deficits caused by variation of 1 RM%.

The variances observed in studies [29–31, 33] may be due to disparities in study methods, and the large variation in CK response within and between studies makes a definitive conclusion on the contribution of intensity and volume of exercise on cell changes difficult. Considering the significant increase in CK levels which have been found as a result of high-intensity exercise compared to lower intensity [29, 30], the decrements in performance experienced [29, 31], and higher levels of PGE_2_ reported [33] even when exercise volume is standardised suggests that higher-intensity exercise will cause the greater disruption of cell membranes; however, with adequate recovery, it may also elicit the greatest adaptations to exercise in the shortest time.

Seven continuous days of the same isokinetic maximal elbow flexion protocol (ECC2 to ECC7) did not increase indices of muscle disturbance compared to a control group who performed only one session of the exercise protocol (ECC1) [27]. Plasma CK levels increased significantly (*P* < 0.05) in both groups, peaking 4 days after the initial bout of exercise. There was a decline in levels over the course of the next 6 days, and both groups had insignificant CK plasma levels at day 7; there was no significant difference between groups at any time. This was attributed to increased resistance to muscle stress or to that no further muscle disruption had occurred [27]. Total work was reduced in the ECC2 to ECC7 group at each of the six further exercise sessions compared to the first day of exercise; however, they were considered to be of the maximal intensity possible, even if at a lower absolute magnitude.

Despite theories of muscle protection and reduced disruption from further consecutive eccentric disruption afforded by the initial exercise bout in this study, the loss of muscle force which resulted in reduced work load presumably would have influenced the results. It is interesting to consider whether the initial loss of CK contributed to the loss of strength over the 6-day period or whether the loss was associated with disruption to type II fibres.

A number of studies have used very high intensity or volume of exercise, or both, to ensure muscle disruption is elicited [34, 35]. Lower-intensity submaximal muscle voluntary contractions (60% MVC) have been shown to be linearly related to CK levels, muscle oedema, and perception of pain compared to higher intensity (80% MVC) [36]. Evans et al. [36] suggest that greater magnitudes of muscle disturbance may alter time course and correlation between muscle fibre damage, pain, and CK release and may in part account for reports by some studies that CK is not a reliable marker of muscle disruption. It has been proposed that, in fact, moderate levels of force may produce superior measurement parameters [35].

## 6. Gender Influences on Muscle Damage

Gender difference in muscle disturbance and repair processes has frequently been reported in the literature. Studies on female animals have demonstrated lower baseline levels of CK and an attenuated CK response to exercise [37, 38]. However, females presented with a higher CK peak and a greater relative increase in serum CK levels after 50 maximal eccentric contractions of the arm flexor muscles, despite significantly lower baseline levels compared to males [39]. Thirty minutes of stepping exercise resulted in a CK serum increase in 15 women from a baseline of  191 ± 103 U/L to  7239 ± 2403 U/L at day 3. There was no significant increase in CK serum levels in the 18 men who performed the same protocol (see Figure 3(c)), however, the authors suggest this may in part be due to greater adaptation to this type of exercise in the males [12].

Rinard et al. (2000) suggest that many of the findings that indicate women have an attenuated response to muscle damaging exercise are due to poor study design and may apply more specifically to aerobic exercise and that there is little or no difference between males and females in their response to eccentric-exercise-induced damage [40]. This view is supported in a review by Clarkson and Hubal (2002) who conclude that any differences between genders are small and indicate that females may be more inclined to muscle disruption than males [41].

In postmenopausal women not taking hormone replacement treatment (HRT) [42] and amenorrheic women [15], raised levels of CK in response to exercise-induced muscle disruption were found, when compared with women on HRT and premenopausal women. This effect was attributed to lower oestrogen levels. Oestrogen may be important in protecting cell membranes from damage [11] and reduced infiltration by leucocytes may lessen their damage causing function in the repair process. Conversely, this may also delay the healing process [43]. Leucocytes may have a role in the activation of satellite cells [11] which proliferate and differentiate forming new muscle fibres [44]. Whether oestrogen can promote reduced CK efflux via reduced membrane permeability or whether actual muscle damage is reduced is not clear [43]. Progesterone has been suggested to interact with oestrogen and may antagonise the oestrogen disruption limiting properties [44].

A study by Arnett et al. (2000) examined CK response to unaccustomed eccentric hamstring exercise in premenarcheal (P) and menarcheal girls (M) and postmenopausal (PM) women. Preexercise levels of CK were significantly greater in PM than in P girls or M women, and CK-MB was greater in M than in both P girls and PM women. However, after exercise M women had significantly higher levels of CK and CK-MB than both P girls and PM women at 24, 48, 72, and 96 hours after exercise. This study concluded that oestrogen levels had no significant effect on CK levels after strenuous eccentric exercise [45]. However, knee ROM in subjects was not assessed. Variations in ROM have been suggested as affecting the mechanical strain on the muscle during eccentric exertion [25]. This activity alters the force applied to sarcomeres and modifies the magnitude of disturbance [46]. Work volume in each group was not measured; therefore, variations between groups may have occurred, affecting associated muscle disruption, and high baseline CK levels in PM may be related to age variations in energetics.

## 7. Age-Related Muscle Disruption

Studies of serum CK response to exercise in aging human skeletal muscle have produced variable results. A review by Fell and Williams (2008) on the effect of aging on skeletal muscle in athletes suggests that aging can lead to greater exercise-induced damage and a slower repair and adaptation response [47]. Muscle mass and function gradually decline with age, and cell apoptosis may have a role in age-related sarcopenia [48]. Lower levels of plasma CK in older female subjects have been attributed to a decline in circulating neutrophils with age which may, in part, be due to reduced oestradiol levels and endogenous antioxidant status [45]. Circulating neutrophils produce oxidants such as superoxide free radicals, which increase cell damage and leakage. Therefore, an increased serum CK could be related to optimal functioning of the cell, which may decline with age, and is not simply a marker of less damage. Free radial production appears to moderate signalling for adaptation of skeletal muscle in response to exercise [49], and this response may be attenuated in older muscle, rendering it less adaptive to exercise stress.

Studies on humans have produced conflicting results in relation to aging muscle response to exercise. Some show evidence of more muscle ultrastructure damage in older subjects (67 ± 3 yrs) compared to young (26±1 yrs) [50] and others in relation to less damage in older (59.4 ± 10.9 yrs) subjects compared to younger (23.4 ± 6.9 yrs) [45]. Lavender and Nosaka (2008) reported no significant changes in indirect measures of muscle damage after unaccustomed eccentric elbow flexion by males (19–25 yrs and 41–57 yrs) [25]. Individual ROM at the elbow was not significantly different between subjects; however, during the exercise the investigator assisted subjects in keeping the velocity of the movement constant. This may have affected the magnitude of muscle damage.

Subjects in this study were described as habitually active. Regular physical activity has been shown to slow the process of sarcopenia and may reverse age-related muscle apoptosis [51]. Exercise may also attenuate and protect against exercise muscle disruption and subsequent damage. Therefore, the level of past and present physical activity may significantly affect muscle damage throughout the ageing process. It would be of interest to explore the effects of habitual training in different age groups and its effect on CK serum levels.

Exposure to exercise stress initiates adaptation in gene expression, cellular protective mechanisms, and remodelling, which help protect muscle during subsequent bouts of exercise [49]. The ability of aged muscle to adapt to environmental stress appears to be impaired, as are repair mechanisms, and heat shock protein (HSP) production is reduced in response to physiological stress in animals [49, 52].

Exercise disturbs muscle homeostasis by depleting glycogen, lowering pH, increasing hyperthermia, and increasing ROS (reactive oxygen species) production as a by-product of energy metabolism. These perturbations (or a combination of them) will initiate a stress response which instigates the release of HSPs such as HSF1 and its cochaperones, for example, HSP70 or HSP90 [53].

In particular, higher levels of ROS after exercise can increase the oxidation of thiol (sulphydryl) groups on proteins, leading to increased protein damage, and may trigger release of HSF1 [54]. Once exercise stress has subsided, cochaperone HSPs bind to HSF1 and deactivate it [55]. The instigation of an HSP response is dependent on a number of factors including the type and intensity of exercise, muscles involved, and the age and training status of the individual. The aging process appears to change ATP pathways, alter muscle fibre type ratios, and reduce HSPs response, which are thought to offer some degree of protection against further exercise-induced muscle damage.

## 8. CK and the AMPK Energy Sensor

**Figure 4:**
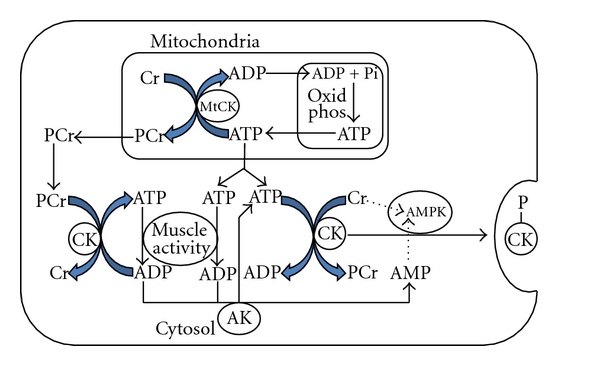
Potential roles of adenylate kinase (AK) and AMP-activated protein kinase (AMPK) in gross control of creatine kinase (CK) activity by promoting expulsion of CK from the cytosol to limit CK utilisation of ATP for PCr resynthesis adapted from Saks [5].

AMPK (AMP-activated protein kinase) is an energy sensing enzyme that is widely dispersed in nature from single-cell organisms to humans, is central to the management of energy supply, and operates both locally and at whole organism (see Figure 4). At times of rest/inactivity it is inactive, and metabolic processes focused on synthesis, storage, and accumulation proceed unhindered. When activities occur that deplete ATP levels, such as physical exercise, glucose depletion, or hypoxia, AMPK is activated. When activated, it in turn stimulates a range of physiological and biochemical processes and pathways that increase ATP production and at the same time switch off pathways that involve ATP consumption. Recent work has shown a strong correlation between a sedentary lifestyle, inactive AMPK, and morbidity diseases such as metabolic syndrome, type 2 diabetes, and dementia [56]. The benefits of exercise in providing protection from such morbidity diseases are now firmly linked to activation of AMPK and associated biochemical and physiological processes that are stimulated. The primary activity of AMPK is to phosphorylate proteins especially enzymes and by this action regulate the activity of key enzymes that operate important reactions and pathways.

The role of CK in energy management is maintenance of PCr levels to provide an immediate energy supply in the first few seconds of physical activity. It is likely that AMPK has a role in controlling CK activity, and some work has demonstrated that AMPK may regulate CK and is sensitive to the Cr : PCr ratio and that increased creatine levels stimulate AMPK activity [57]. Given the widespread action of AMPK (during exercise) to switch off nonessential ATP consumption, it is likely that AMPK would act to limit the use of ATP by CK to produce PCr and re-establish the PCr pool [58]. During intense exercise there is no PCr resynthesis and the reaction is likely blocked by more than one mechanism; however, although there is no need for PCr resynthesis, there is a need to maintain the ratio and AMPK could be part of the overall process.

It is clear that such a system would not act in isolation but as part of a sophisticated process involving other regulatory functions in the muscle, and only when the full integrated system is understood will it be possible to explain the many anomalies associated with muscle action. For example eccentrically biased exercise (e.g., downhill running) will elicit greater postexercise levels of serum CK than equivalent concentrically biased exercise (e.g., uphill running) though the former is less energy metabolism demanding than the latter [41]. This highlights the integrated complexity of metabolism and mechanical damage as eccentric-biased exercise is associated with increased indices of muscle damage (i.e., DOMS) which is mainly a result of micro-damage within the myocyte [59, 60]. In addition, eccentric-biased contractions may be more mechanochemically efficient based on changes in the actin-myosin sliding length in that changes in sliding length produce different levels of tension and consequent different degrees of muscle damage [61]. It may be that as eccentric and concentric contractions have different demands and consequences on the metabolic and mechanical components of muscle action, there are alternate mechanisms of control via AMPK that produce different effects of CK levels. This would allow maximum flexibility for a wide range of exercise stressors to enable survival of the species in prehistoric environments where survival depended upon adaptable and flexible muscle action.

ATP levels never deplete to critical levels; this is because the sensitivity of ATP is set very high to guarantee that they never deplete, so a slight reduction in high ATP level triggers an early protective reaction. It is postulated here that in some circumstances, either directly or indirectly, the controlling activity of AMPK could trigger a process that culminates in the elimination of CK from the cell as part of a mechanism to regulate metabolic and/or mechanical disruption of muscle cells to prevent muscle failure caused by accumulative damage with consequent increase in serum CK levels (see Figure 4). This might be a component function in the overall action of fatigue to limit muscle activity or it could be a system that evolved prior to or in parallel with fatigue mechanisms.

The AMPK mechanism of control involves phosphorylation of CK, and it may be that phosphorylation provides a signal to facilitate removal of CK from the cytosol (see Figure 4). Such a mechanism would explain the appearance of serum CK following physical exercise as opposed to structural damage arising from muscle trauma. Following muscle damaging exercise, CK levels continue to rise in the blood for hours or days (see Figures 3(a)–3(c)) despite significant metabolic disruptions having ceased. The capacity for compromised muscle tissue to generate force is impaired [27, 31, 62]; therefore, measures are required to protect and facilitate the repair of muscle tissue. In addition, other processes which disrupt the cell membrane, for example, inflammation, continue [63], allowing CK to exit the cell over time. This extended loss of CK may be associated with protective mechanisms, and a prolonged involvement of AMPK, allowing repair and restoration of muscle function.

## 9. Influence of Genetic Characteristics

Exercise-induced muscle disruption is known to produce insulin-like growth factor II (IGF II) in response to cell damage and is thought to stimulate satellite cells and hypertrophy. An association has been found between a polymorphism in the sarcomeric protein myosin light chain kinase and changes in blood CK, Mb, and isometric strength, in individuals with specific genetic variations in alleles of IGF II who experienced increased muscle disruption as a result of maximal isotonic eccentric contractions [64]. This suggests that these genome variations may lead to alterations in calcium handling and force effects during exercise, thereby influencing muscle disruption. This could explain the susceptibility of some individuals, who are otherwise healthy, to muscle disruption and exertional rhabdomyolysis [64] and the large intersubject variation in levels of serum CK found in many studies.

Heled et al. (2007) explored the possibility of a genetic association between CK MM, angiotensin-converting enzyme (ACE) genotypes, and CK response to exercise [7].

 A genetic association was found between a specific CK-MM genotype of the *Ncol *polymorphism with an augmented response to exercise.

Yamin et al. (2007) found an association between type of ACE genotype and CK levels. ACE genotypes may be involved in the excitation coupling process and influence the risk for developing rhabdomyolysis and, conversely, protection against exercise-induced muscle injury. However, this effect may be more noticeable in previously sedentary individuals performing intense exercise [65]. Other studies featuring physically active subjects did not find a comparable association [7].

Intensive exercise initiates an immune response resulting in acute and delayed leukocytosis, featuring neutrophils predominantly. It develops approximately 30 minutes after acute exercise, and leukocytosis peaks several hours after exercise before returning to baseline levels 24 hr after exercise [66]. This delayed proinflammatory response may in part be related to the serum CK response observed after exercise-induced muscle damage, due to leucocytes infiltrating and destabilising the cell membrane during the process of repair.

Serum CK followed a biphasic pattern increasing until 23 hr after exercise declining weakly at 47 h before increasing again and peaking 95 h after exercise. This biphasic response has been noted in other studies [23, 35] and may be related to the time line of inflammation.

## 10. Exercise Modality

Exercise modality can affect the appearance of CK in blood serum. Eccentric resistance training CK serum levels can peak between 72 hrs [31, 45] and 96 hrs [67] to 120 hrs [4] (see Figure 3(b)). Training status may affect this time response. Full body eccentric resistance training in resistance trained (RT) and untrained (UT) men elicited a significant (UT *P* = 0.002, RT *P* = 0.02) increase in CK serum levels at 24 hrs. This signified the peak response in the RT group, whilst levels in the UT group continued to rise and peaked at 72 hrs [68]. However, three sets of 50 maximal eccentric leg flexion contractions in untrained men resulted in a significant (*P* < 0.05) increase in CK serum levels at 24 hrs; levels decreased over the next 2 days followed by a nonsignificant (*P* > 0.05) increase at 96 hr [23], and 10 sets of 10 reps of 70% body mass barbell squats incorporating eccentric and concentric contractions in non-resistance-trained males and females resulted in a peak serum CK response at 24 hr after exercise. A series of plyometric jumps performed over 2–5 minutes by untrained men produced a peak CK serum response at 48 hrs [69], and 90 minutes of endurance cycle ergometer exercise at a set absolute workload (1.5 kilo ponds at 60 revolutions per minute) performed by untrained men three days consecutively caused a significant (*P* < 0.05) increase in serum CK levels 3 hours after the first exercise session and peak CK serum levels occurred immediately after the third day of exercise, 72 hrs from the initiation of exercise [6] (see Figure 3(a)). Stepping exercise resulted in a CK serum increase in women at day 3, whereas, there was no significant increase in CK serum levels in men performing the same protocol (see Figure 3(c)).

Pantoja et al. [70] analysed the muscle disruption effect of dynamic resistance training performed on land or in water. The duration of the ten-rep max for elbow flexion for each subject was recorded with a chronometer in order to standardise exercise in both land and water environments and induce the same energy-generating metabolic pathways. Subjects executed as many maximal effort contractions as possible for each set performing three sets in both environments with two-minute rest between sets; each environment session (land or water) was separated by four weeks.

A significant increase in serum CK was observed at 48 hours after exercise on land, and no significant change in baseline serum CK levels occurred in water. No further samples were taken after this time. The main mechanism hypothesised to have attenuated muscle damage in water was reduced eccentric contractions [70].

There are difficulties in comparing exercise intensity and work volume in land and water [71, 72]. Standardisation of exercise between water and land is challenging due to the differing conditions in water compared to air (resistance, temperature, and hydrostatic pressure).

The significance of exercise modality on CK serum response appears to be related to the magnitude of eccentric contractions involved in the activity and the subsequent extent of muscle disruption. Greater muscle cell disturbance delays the appearance of a CK serum peak compared to less disruption. This may be linked to the time course of inflammation; however, evidence in the literature supporting this theory remains unclear.

## 11. Conclusion

The molecular mechanisms that result in CK release from muscle after mild exercise are unclear. More clarification could provide important information for athletes concerned about muscle hypertrophy, performance, and the importance of rest periods between periods of exercise. Future studies should include an exploration of ethnic variations in CK response to exercise. In the absence of any mechanical muscle damage, it remains a question as to whether raised CK after exercise does represent a degree of actual muscle damage or some form of disruption in energy control processes or some other molecular reaction mechanism.

Since muscle tissue cannot ignore brain centred nerve stimulations causing increase in both the number of motor units recruited and the frequency of motor unit stimulation, as well as creation of longer tetanic contractions, it would seem logical that muscle would have some mechanism of moderation to delay the final sanction of fatigue for as long as possible. It is considered here that this could be a membrane event in which a proportion of cytoplasmic enzymes/proteins such as CK exit the muscle cell to place a temporary energy restriction and allow subsequent relaxation and regeneration.

A key regulator in this event would be the energy sensor enzyme AMPK, which can phosphorylate CK and is sensitive to Cr/PCr ratios. At the start of physical exercise the initial supply of ATP for muscle activity is provided via the Cr-PCr shuttle by the readily reversible CK catalysed conversion of PCr + ADP to Cr + ATP until PCr is depleted. As physical activity continues and ATP is increasingly produced by oxidative phosphorylation, there is potential for the rapid rise in ATP levels to be blunted if both MtCK and cytosol CK use the ATP to regenerate PCr. Since AMPK has an overall role, during physical exercise, to limit ATP consumption by nonessential systems, it is likely that this extends to CK. Although PCr resynthesis is greatly diminished during high-intensity exercise, AMPK may still be required to maintain the ratio. It is speculated here that the control involves expulsion of CK from the cytosol (see Figure 3). If this is the case, then increased serum CK levels arising from normal physical exercise may be a consequence of normal metabolic activity rather than representative of physical damage to muscle. Further the wide ranges of serum CK found in the population could reflect different levels of sensitivity of AMPK and/or levels of AMPK, resulting in varying levels of control and hence varied expulsion of CK from the cytosol. Such a system would not act in isolation but as part of a sophisticated process involving other regulatory functions in the muscle, and only when the full integrated system is understood will it be possible to explain the many anomalies associated with muscle action.

It is suggested here that the appearance of CK in serum following low- to moderate-intensity exercise represents a disturbance to muscle energy processes and is not representative of the type of muscle cell damage observed following MI, stroke, or other physical/structural damage. Unfortunately, it has not been possible from the available literature to extract more definitive evidence for this suggestion. The considerable variability across many studies makes interpretation more difficult, and it is clear that the lack of agreed guideline procedures and defined parameters for the conduct and evaluation of exercise-based experimental work in this area is a major barrier to the greater understanding of the influence of exercise on muscle and human health in general. The establishment of an international committee on exercise-based experimental and laboratory protocols may be beneficial. Such a committee could provide leadership, clarity, and standardisation that would enable researchers to effectively answer related experimental questions.

## Figures and Tables

**Table 1:** Some conditions causing or contributing to rhabdomyolysis adapted from Huerta-Alardín et al. with permission from BioMed [10].

Causes of Rhabdomyolysis	
Physical causes	Examples
Trauma/compression	Crush injury, motor vehicle trauma, bed-confinement, physical torture and abuse, and prolonged surgery
Vessel occlusion	Embolism, thrombosis, limb tourniquet, or clamp
Shock	Drug overdose
Excessive muscle activity	Epileptic fit, overexertion (marathon running), tetanus
Electric current	Electrocution, cardioversion, and lighting strike
Hyperthermia	Prolonged exercise in hot climate, infections, and cancer

Nonphysical causes	Examples

Metabolic dysfunction/disturbance	Carnitine deficiency, various enzyme deficiencies, and mitochondrial and electron transport disturbance
Toxins	Heavy metals, venoms
Drugs and chemicals	Antipsychotics, antidepressants, hypnotics, narcotics alcohol, halothane, laxatives, salicylates, and strychnine
Infections	Viruses, bacteria, fungi, and parasites
Electrolyte imbalance	Hyperosmosis, hyper-/hyponatremia, hypocalcaemia, and hyper/hypokalemia
Endocrine disorders	Ketoacidosis, hyper-/hypothyroidism, and diabetes mellitus
